# 

**Published:** 1862-10

**Authors:** 


### WORKS PUBLISHED DURING THE QUARTER.

### 1. *Medicine.*

Aitken.—On the Growth of the Recruit and Young Soldier, with a view to a judicious Selection of "Growing Lads" for the Army. Fcap. 8vo, limp cloth, 2s. 6d.

Althaus.—The Spas of Europe. By Julius Althaus, M.D. 8vo, cloth, 12s.

Blake.—Defects in the Moral Treatment of Insanity in the Public Lunatic Asylums of Ireland, with Suggestions for their Remedy, and some Observations on the English Asylums. By John A. Blake, M.P. 8vo, 1s.

Braithwaite.—Retrospect of Medicine, January to June, 1862. Vol. XLV., fcap. 8vo, cloth, 6s.

Davy.—On some of the more important Diseases of the Army, with Contributions to Pathology. By John Davy, M.D., F.R.S. 8vo, cloth, 15s.

Gairdner.—Clinical Medicine; with Commentaries. By W. T. Gairdner, M.D., &c. Post 8vo, cloth, 12s. 6d.

Hints for Clinical Clerks in Medical Cases. Fcap. 8vo, 1s.

Haughton.—The Theory of Vital Force applied to the Cure of Disease. By E. Haughton, A.B., M.D., M.R.C.S.E. 8vo, 1s.

Jones.—Self-Supporting Dispensaries: their Adaptation to the Relief of the Poor and Working Classes, with Directions for the Establishment and Management of such Institutions. By John Jones, M.R.C.S. 8vo, 1s.

Lee.—Vichy and its Mineral Springs. By Edwin Lee, M.D. Fcap. 8vo, 1s.

Murphy.—Principles and Practice of Midwifery. New and improved edition, with many Illustrations. By E. W. Murphy, M.D., &c. One Vol. small 8vo, 12s. 6d.

Pratt.—On Eccentric and Centric Force: a New Theory of Projection. By Henry F. A. Pratt, M.D. 8vo, cloth, 10s.

Price.—The Winter Climate of Menton; with Hints to invalids intending to reside there. By P. C. Price, F.R.C.S.E. Fcap. 8vo, cloth, 3s.

Ranking.—The Half-Yearly Abstract of the Medical Sciences. Edited by W. H. Ranking, M.D., and C. B. Radcliffe, M.D. Vol. XXXV., price 6s. 6d.

Silvester.—The Physiological Method of Treating Consumption. By Henry R. Silvester, B.A., M.D. London. 8vo, 1s.

Smee.—General Debility and Defective Nutrition: their Causes, Consequences, and Treatment. By Alfred Smee, F.R.S. Second Edition, fcap. 8vo, cloth, 3s. 6d.

### 2. *Surgery.*

YEARSLEY.—Introduction to the Art of Laryngoscopy: a new Method of Diagnosing Diseases of the Throat and Larynx. By James Yearsley, M.D. 1s., with Woodcuts.

HOLMES.—A System of Surgery, Theoretical and Practical, in Treatises by various authors. Edited by T. Holmes, M.D. Vol. III., Operative Surgery—Diseases of the Organs of Special Sense, Respiration, Circulation, Locomotion, and Innervation. 8vo, cloth, 21s.

MILTON.—On Spermatorrhœa and its Complications. By J. L. Milton, M.R.C.S. Sixth Edition, 8vo, sewed, 2s.

PARKIN.—The Utilization of the Sewage of Towns. By John Parkin, M.D. 8vo, 2s.

---

### FOREIGN MEDICO-PSYCHOLOGICAL LITERATURE.

OUR Retrospect of current Foreign Medico-Psychological Literature will embrace the following subjects:

1. The Relations of Inebriety to Insanity.

2. Cases of Fracture of the Ribs in Insane Patients discovered after Death.

3. On Moral Insanity in Relation to Criminal Acts.

### INDEX TO VOL. II.

Aërophopia, 564
America, the Civil War in, 722
——— sanitary condition of Federal army, 337, 613
——— ——— after retreat, 722
——— sanitary commission of United States, 613
——— effects upon insanity, 744
Anthropophagy, 711
Army, the health of, 230
——— suicide in, i.
Australia, lunacy reform in, 720
Asylums, on the organization of, 176
——— French metropolitan, proposed reforms in, 592

Belloc, Dr., on lesion of association of ideas, 169
Boismont, B. de, on hallucinations in relation to medical jurisprudence, 64
——— on hallucinations in insanity, 283, 523
British Medical Association, 30th meeting of, 718

Chabas on the antiquity of plague, 554
——— on the medicine of the ancient Egyptians, 725
Charles II., Dr. Chevers on cause of death of, 155
Cholera in India, 152
Clarke, George, the case of, 304
Clarke, Mr. J. Lockhart, on Volition, 570
Cook, Dr., on Inebriety and Insanity, 734
Counter practice, 443
Coxe, Dr., on Gheel, 184
Crimes, extraordinary, ii.
Curwen, Dr., Diagnosis of certain forms of Insanity, 743

Davies, Rev. W. S., on the province of psychology, 255
——— on mental suggestion, reproduction, and association, 639
Dream-thought and dream-life, 193
Drunkenness, Salvatori on, 483
——— and Insanity, 734

Eccentricity and capital charges, xxv.
Education; on New Minute of Council, 113
Egyptians, ancient, medicine of, 725
England, state of lunacy in, 667
Exhibition, the International, 424

"Fact," the legal doctrine of, and the case of George Clark, 304
Falret, Dr. Jules, report on Gheel, 364
Female Medical College, 546
Fleming, Dr., on insane institutions and colonies, 360
French metropolitan asylums, reform in, 592

George III., Earl Stanhope's account of mental malady of, 367
Gheel, a recent visit to, by Dr. Coxe, 184
——— report of a commission of Medico-Psychological Society of Paris on, 364

Hallucinations in relation to medical jurisprudence, 64
——— in insanity, 283, 523
Hastings, Dr., *v. The Lancet*, 551
Herbert, Lord, memorial to, 153, 338
Hospital, St. Thomas's, removal of, 334, 549

Hospitals, English and French, comparative merits of, 339

IDEAS, on lesion of association of, 169
India, cholera in, 152
Insane institutions and colonies, 357
Insanity, certain forms of, not suited to an asylum, 564
——— and common juries, xxii.
——— and inebriety, 734
——— diagnosis of certain forms of, 743
——— influence of civil war on, 744
——— undetected fractured ribs in, 738
Instinct and reason, 12
Ireland, state of lunacy in, 102
——— registration of births and deaths in, 341

KLEPTOMANIA, case of, 357

LUNACY, state of, in Ireland, 102
——————— England, 667
——————— Scotland, 681
Lunacy reform in Australia, 720
Lunatics, murders by, iv., xvi.
——— colonization of, by the Legislature, 433

MACWILLIAM, Dr., death of, 554
Marvellous, The, the *Times* on, xii.
Mauthner, Dr., on a case of kleptomania, 357
Medical Council, the, 1, 546
Mental suggestion, association, and reproduction, 639
Micropsychology, 131
Murder, epidemic of, xviii.
——— in the army, i.
——— by children and youths, ii.
——— by lunatics, iv., xvi.
——— and suicide, ii.

NAVY, Royal, health of, 473

PERIODICITY, 37
Plague, antiquity of, 554
Poisoning, slow and secret, 265
Poisons, the sale of, xv.
Poison, a wonderful, xiv.
Prince Consort, the death of, 168
Psychology, the province of, 255
——— the debateable land in, 507

QUARANTINE, report of Social Science Association on, 84
——— note on, 380
——— in the Thames,

RADCLIFFE, Mr., on Suicide, 701
Renaudin, Dr., on the organization of a lunatic asylum, 176
Romantic psychology; a Strange Story, 456
Rumball, Mr. J. Q., on instinct and reason, 12

REVIEWS AND BIBLIOGRAPHICAL NOTICES.

Althaus, Dr., on the Spas of Europe, 730
Anderson, Dr. T. M'Call, on Parasitic Affections of Skin, 164

Bigg, Mr., on Mechanical Appliances in treatment of Deformities, 727
Blake, Mr. J. A. (M.P.), Defects in Irish Asylums, 732
Brodie, Sir B., Psychological Inquiries, 2nd part, 731
Brown, Mr. Baker, on Ovarian Dropsy, 732
Bulwer's, Sir E. B. Lytton, Strange Story, 456

Catlin, Mr., the Breath of Life, 351
Chuckerbutty, Dr., on Native Education in India, 36
Collyns, Mr., Chase of Wild Red Deer, 343

Davies, Rev. W. G., the A, B, C, of Thought, 556
Dowson, Dr., on Erasmus Darwin, 562

Epidemiological Society, Transactions of, 351

Fleischmann, Mr. A., Practical Precepts for Plain People, 355

Gilbart, Mr., Logic for the Million, 165

Hulke, Mr., on the Ophthalmoscope, 159
Hunt, Mr., Diseases of the Skin, 164

McIntosh, Dr. Carmichael, on Carcinus Mœnas, 165
McWilliam, Dr., on Health of Merchant Seamen, 354
Martin, Sir J. R., on Tropical Diseases, 347

Medical Society of London, Transactions of, 165
Miller, Professor, on Neuenahr, 731
Munk, Dr., Roll of College of Physicians, 158

Ordronaux, Dr., Hints on Preservation of Armies, 166

Pain, Dr. de l'Hygiène Morale de la Folie, 161
Parigot, Dr., Moral Insanity in Relation to Criminal Acts, 740

Richardson, Dr., Clinical Essays, 559
Reynolds, Dr., on Epilepsy, 347

Smith, Dr. Ed., Cyclical Changes in Health and Disease, 37
——— on Consumption, 728
Stokes, Dr., Influence of Civil War on Insanity, 744
Sydenham Society, Selected Monographs, 163
——— Casper's Forensic Medicine, 164

Tuson, Mr., on Spinal Debility, 163
Thompson, Mr. Henry, on Diseases of Prostate, 164

Wheatley, Mr. H. B., on Anagrams, 561
Witt, Mr. C., on an Effectual Remedy for Scarlet Fever, 354

Salvatori, on drunkenness, 483
Scotland, lunacy reform in, 331
——— state of lunacy in, 681
Session of 1861-62, Opening of, 147
Small-pox among sheep, 684
Social Science, National Association of, xvii.
Spirit mediums, ix.
Stanhope, Earl, on mental malady of George III., 367
Stanley, Mr., death of, 554
Street-folk, the, 120
St. Thomas's Hospital, removal of, 334, 549
Suicide in the army, i.
——— æsthetics of, 379
——— restraint of, 701
Suicide-fields, 701

United States, sanitary commission of, 613

Vapours, noxious, 656
Variola ovina, 684
Vivisection, protest against, 154
Volition, on the nature of, by J. Lockhart Clarke, 570
Volunteers, sanitary condition of United States, 613

Solomon, Dr., on aërophobia,
Windham, the case of Mr. W. F., 381
Works published, 166, 355, 562, 733
Workman, Dr., cases of undetected fractured ribs in the insane, 738

END OF VOL. II.

